# Antileishmanial potential of polar fractions from *Amaranthus blitoides* S. Watson, *Caucalis platycarpos* L., and *Cistanche flava* (C.A.Mey.) Korsh.: evidence from in vitro and in vivo studies

**DOI:** 10.1186/s41182-026-01076-5

**Published:** 2026-10-12

**Authors:** Mohadese Miri, Ardalan Pasdaran, Mohammad Saleh Bahreini, AmirReza Dehghanian, Bahador Sarkari, Naghmeh Dastan, Faeze Karimi, Kambiz Karimi, Negin Javanmardi-Nia, Mohammad Hossein Motazedian

**Affiliations:** 1 Department of Parasitology and Mycology, School of Medicine, Shiraz University of Medical Sciences, Shiraz, Iran; 2 Department of Pharmacognosy, School of Pharmacy Medicinal Plants, Shiraz University of Medical Sciences, Shiraz, Iran; 3 Basic Sciences in Infectious Diseases Research Center, Shiraz University of Medical Sciences, Shiraz, Iran; 4 Department of Pathology, School of Medicine, Shiraz University of Medical Sciences, Shiraz, Iran; 5 Department of Parasitology and Mycology, School of Medicine, Hamedan University of Medical Sciences, Hamedan, Iran; 6 Department of Immunology, School of Medicine, Shiraz University of Medical Sciences, Shiraz, Iran

**Keywords:** Cutaneous leishmaniasis, *Amaranthus blitoides* S. Watson, *Caucalis platycarpos* L., *Cistanche flava* (C.A.Mey.) Korsh., *Leishmania major*, In vitro, In vivo

## Abstract

**Background:**

Cutaneous leishmaniasis (CL) is recognized by the World Health Organization (WHO) as a research priority, yet its treatment remains challenging. CL lesions arise from complex interactions between the parasite and host immunity, where excessive inflammation can delay healing. Given the anti-inflammatory and immunomodulatory properties of medicinal plants, this study evaluated the effects of polar fractions from methanolic extracts of *Amaranthus blitoides* S. Watson*, Caucalis platycarpos* L., *Cistanche flava* (C.A.Mey.) Korsh., and their mixtures on CL caused by *Leishmania major*.

**Methods:**

In vitro, the antileishmanial activity of polar fractions against *L. major* promastigotes and their cytotoxicity on RAW 264.7 macrophages were assessed using flow cytometry and MTT assay, respectively. In vivo, female BALB/c mice infected with *L. major* were treated with polar fractions. Lesion size was monitored weekly, and at the end of treatment, parasite load at lesion margins and seven liver biochemical parameters in serum were measured.

**Results:**

Polar fractions exhibited mild-to-moderate antileishmanial effects on promastigotes without significant cytotoxicity to macrophages. In vivo, treatment with 20 mg/kg *C. platycarpos*, 20 mg/kg *C. flava*, and 80 mg/kg of the mixture significantly reduced lesion size compared to controls. Clinical observations also indicated reduced local inflammation in these groups. Parasite load remained detectable across all groups, and liver biochemical parameters did not differ significantly from the negative control.

**Conclusion:**

Polar fractions of the studied plants demonstrated safety and therapeutic promise, supporting their potential as adjunctive candidates for further investigation in the management of CL caused by *L. major*.

## Introduction

Cutaneous leishmaniasis (CL) is a parasitic disease caused by the entry of obligate intracellular protozoa of the genus *Leishmania* into the skin, a process initiated by the bite of an infected sandfly [1]. *Leishmania major* is the primary species responsible for CL in humans and animals in the Old World [2]. The disease manifests as skin lesions with localized inflammation and remains highly prevalent worldwide [3]. In addition to causing skin lesions, CL is associated with a complex, multi-step immune response [4]. To effectively control the infection, the immune system must be finely tuned, as any disruption of this delicate balance can alter the disease course [4]. On one hand, the appropriate production of inflammatory cytokines is essential for inhibiting parasite proliferation; on the other hand, an excessive increase in these molecules can lead to massive recruitment of immune cells to the lesion site [5]. This intense cellular accumulation not only exacerbates local inflammation but may also result in tissue destruction and lesion deterioration [6]. Furthermore, *Leishmania* parasites employ various mechanisms to circumvent the host's immune response, thereby creating favorable conditions for their survival [7]. This complex interplay between the parasite and the immune system constitutes a major reason for the treatment difficulties and persistence of skin lesions in CL [8, 9]. Current treatments, including drugs such as meglumine antimonate, amphotericin B, and miltefosine, primarily target the parasite itself, yet they often leave disfiguring scars that carry significant economic and psychological impacts [10]. Moreover, these treatments are associated with considerable side effects [10, 11]. Such limitations have shifted attention toward natural alternatives, particularly medicinal plants. Medicinal plants and their bioactive derivatives have garnered significant interest as complementary strategies for wound healing and treatment. Global reviews conducted between 2020 and 2024 provide consistent evidence that phytochemicals—including flavonoids, phenolic acids, terpenoids, and glycine derivatives—can serve as antibacterial, antioxidant, antimicrobial, and wound-healing agents in herbal medicine. These compounds exert their therapeutic effects through mechanisms such as angiogenesis, enhanced wound contraction, and re-epithelialization in preclinical models. Topical application of plant derivatives offers promising prospects for tissue repair in leishmaniasis lesions [6, 12–15]. Given the importance of obtaining specific bioactive compounds, selecting the appropriate solvent plays a pivotal role, as solvent type directly influences the quality, quantity, and profile of bioactive compounds extracted. The use of polar solvents such as methanol and water typically yields richer extraction of polyphenolic and flavonoid compounds, which are known to play important roles in antioxidant and anti-inflammatory activities. Polar fractions generally contain substantial amounts of these secondary metabolites. Flavonoids, for instance, can modulate inflammatory pathways by neutralizing free radicals and reducing oxidative stress, thereby contributing to an effective therapeutic response. Consequently, the choice of solvent and targeted access to these bioactive compounds are critical factors in maximizing the therapeutic potential of plant extracts [16]. Based on this rationale, the present study evaluated the effects of polar fractions derived from methanolic extracts of *Amaranthus blitoides* S. Watson, *Caucalis platycarpos* L.*, Cistanche flava* (C.A.Mey.) Korsh., and a mixture of all three polar fractions on skin lesions caused by *L. major*. Previous studies have demonstrated that *Amaranthus* species possess antioxidant, anti-inflammatory, and antimicrobial activities [17, 18]. Several recent investigations and reviews have reported that *Cistanche* species exhibit antioxidant, anti-inflammatory, immunomodulatory, and hepatoprotective effects [19–22]. Additionally, *C. platycarpos* has shown anti-inflammatory and antioxidant properties [23, 24]. Collectively, these data provide a pharmacological rationale for employing polar fractions of these plants in CL treatment, leveraging their anti-inflammatory, antioxidant, and wound-healing potential. Accordingly, in this study, we evaluated the efficacy of each polar fraction individually—from methanolic extracts of *A. blitoides*, *C. platycarpos*, and *C. flava*—as well as their combination, each formulated as a topical ointment. A combined in vitro approach utilizing flow cytometry and the MTT assay was employed to investigate the antileishmanial activity and cytotoxicity of the polar fractions, respectively. Furthermore, in vivo studies were conducted to assess changes in skin lesion size using digital calipers and to quantify parasite load via biopsy specimens obtained from the lesion margins in inbred BALB/c mice infected with CL caused by *L. major*. To evaluate potential systemic toxicity of the polar fractions, seven liver biochemical parameters—including total protein (TP), albumin (Alb), alkaline phosphatase (ALP), aspartate aminotransferase (AST), alanine aminotransferase (ALT), total bilirubin (T. Bili), and direct bilirubin (D. Bili)—were measured in serum.

## Materials and methods

### Preparation of plant extracts and their polar fractions

While these plants are commonly recognized as invasive herbs, a thorough review of various references was conducted to assess any potential for endangerment. All species were checked against the IUCN Red List of Threatened Species. *C. platycarpos* and *A. blitoides* were gathered in May 2016 from the Saravan rural district, specifically at Saqalaksar Lake (coordinates: 37.2094, 49.594967) in Guilan province, Iran. Meanwhile, *C. flava* was collected in April 2024 from Nayband National Park in Bushehr province, Iran (coordinates: 27.333099, 52.661598). For all species, voucher specimens were verified by Dr. Ardalan Pasdaran at the Medicinal Plants Processing Research Center, Shiraz University of Medical Sciences in Shiraz, Iran, with voucher specimen numbers 2585, 2589, 2600. These specimens are deposited in the Herbarium of Medicinal Plants Processing Research Center, Shiraz University of Medical Sciences in Shiraz, Iran. The collected aerial parts of *A. blitoides*, *C. platycarpos*, and *C. flava* were dried at room temperature. For each species, 10 kg of the dried plant material was sequentially extracted with 30 L of hexane, 30 L of dichloromethane, and 30 L of methanol, respectively, at room temperature for 48 h, in order of increasing polarity. The resulting methanolic extract from each plant was then dissolved in 5 L of distilled water and fractionated by liquid–liquid partitioning using 15 L of petroleum ether, 15 L of dichloromethane, and 15 L of ethyl acetate, respectively, in order of increasing polarity, and the obtained aqueous fractions (200 g from each species) were dried by spray drying and subsequently used for biological assays [25, 26]. All solvents were of analytical grade and purchased from Sigma-Aldrich (St. Louis, MO, USA). Additionally, to evaluate the effect of the polar fraction mixture, equal amounts (1:1:1, w/w) of the final polar fractions were combined and homogenized.

### Parasite and macrophage cell culture

*Leishmania major* promastigotes (MRHO/IR/75/ER) were obtained from the Department of Parasitology and Mycology, Shiraz University of Medical Sciences, Shiraz, Iran, and were cultured in RPMI 1640 medium (Bio Idea, Tehran, Iran) supplemented with 10% fetal bovine serum (FBS) (Gibco, Waltham, MA, USA) and 1% penicillin–streptomycin (Pen-Strep) (Gibco, Waltham, MA, USA), and incubated at 25 °C. The RAW 264.7 macrophage cells (IBRC-C10072) were obtained from the Department of Parasitology and Mycology, Shiraz University of Medical Sciences, Shiraz, Iran, and cultured in RPMI 1640 medium supplemented with 10% FBS and 1% Pen-Strep, and incubated at 37 °C in 5% CO₂ (NAPCO, Waltham, MA, USA).

### Preparation of polar fraction concentrations of methanolic plant extracts

The antileishmanial activity and cytotoxicity of the polar fractions were evaluated at concentrations ranging from 1 to 512 μg/mL and 1 to 1024 μg/mL, respectively. For this purpose, 10.24 mg of each polar fraction and their mixture were dissolved in 10 mL of phosphate-buffered saline (PBS) under sterile conditions. Serial dilutions were then prepared to obtain final concentrations of 1, 2, 4, 8, 16, 32, 64, 128, 256, 512, and 1024 μg/mL.

### Evaluation of antileishmanial activity by flow cytometry using propidium iodide staining

To evaluate antileishmanial activity, 150,000 promastigotes (500 μL) were transferred to 1.5-mL microtubes and treated with polar fractions at concentrations ranging from 1 to 512 μg/mL (final volume: 1 mL), followed by incubation at 4 °C for 3 h. After incubation, propidium iodide (PI; Sigma-Aldrich, St. Louis, MO, USA) was added to the suspensions at a final concentration of 50 μg/mL and incubated for 30 min in the dark. In addition, one microtube containing promastigotes and PI was used as a negative control, one microtube containing promastigotes and PI with 20 μg/mL amphotericin B as a positive control, and one microtube containing promastigotes without PI was used for flow cytometry calibration. The suspensions were then transferred to polystyrene tubes and analyzed using a FACS Calibur flow cytometer (Becton Dickinson, San Jose, USA) to determine the antileishmanial effects of the polar fractions on *L. major* promastigotes [27].

### Cytotoxicity assessment by MTT assay

To evaluate cytotoxic effects, RAW 264.7 macrophages were treated with polar fractions at concentrations ranging from 1 to 1024 μg/mL, and cell viability was assessed by MTT assay. Briefly, 100 μL of RPMI 1640 medium supplemented with 10% FBS and 1% penicillin–streptomycin containing 30,000 macrophage cells was added to each well of 96-well plates. Then, 100 μL of each polar fraction at concentrations of 1 to 1024 μg/mL was added to the wells. All experiments were performed in triplicate. For each polar fraction, wells containing 100 μL of culture medium and 100 μL of the respective concentration (1 to 1024 μg/mL) served as the control group. In addition, three wells containing only 200 μL of culture medium and three wells containing 200 μL of culture medium with macrophages were included as controls. The plates were incubated for 48 h at 37 °C in 5% CO₂. After incubation, 20 μL of MTT solution (5 mg/mL in PBS) was added to each well, and the plates were incubated for an additional 4 h at 37 °C in 5% CO₂ in the dark. The reaction was then stopped by adding 100 μL of acidic isopropanol to each well. The plates were shaken for 20 min at 25 °C, and the optical density (OD) was measured at 570 nm using an ELISA reader [27, 28].

### Animals and infection protocol

In this study, 70 female BALB/c inbred mice aged 4–6 weeks and weighing 18–20 g were obtained from the Royan Institute (Tehran, Iran). The animals were maintained under standard conditions (temperature 22 ± 2 °C, humidity 50 ± 10%, free access to food and water) at the Laboratory Animal Care Center of Shiraz University of Medical Sciences. To induce CL, *L. major* amastigotes were extracted from the skin lesions of infected mice and suspended in sterile physiological saline. To maintain sterile conditions and prevent microbial contamination, 1% penicillin–streptomycin was added to the suspension, and 0.2 mL of the suspension containing approximately 5000 live amastigotes was injected subcutaneously at the base of the tail. The clinical status of the animals was continuously monitored, and treatment was initiated immediately upon lesion appearance. All experiments were performed in accordance with institutional ethical guidelines and were approved by the Ethics Committee of Shiraz University of Medical Sciences (approval code: IR.SUMS.AEC.1404.078).

### Experimental design and treatment protocol

To evaluate therapeutic effects, topical ointments containing the polar fractions of *A. blitoides*, *C. platycarpos*, and *C. flava* individually and as a mixture were formulated at concentrations of 80, 40, and 20 mg/kg using water and eucerin. These formulations were applied topically to the skin lesions of mice once daily for 21 consecutive days. Infected mice were randomly divided into 14 groups (*n* = 5 per group). Groups 1 to 3 were treated topically with concentrations of 80, 40, and 20 mg/kg of *A. blitoides*, respectively; groups 4 to 6 with concentrations of 80, 40, and 20 mg/kg of *C. platycarpos*, respectively; groups 7 to 9 with concentrations of 80, 40, and 20 mg/kg of *C. flava*, respectively; and groups 10 to 12 with concentrations of 80, 40, and 20 mg/kg of the polar fraction mixture, respectively. Group 13, as a negative control, received only the base ointment (water and eucerin) topically, and group 14, as a positive control, received topical treatment with nanoliposomal amphotericin B (Sina Ampholish gel 0.4%). The experimental unit was a single mouse.

### Monitoring and measurement of skin lesions

Lesion monitoring was performed at five time points: day 0 (pre-treatment), days 7, 14, and 21 (during treatment), and day 28 (one week post-treatment) [29, 30]. Lesion size was measured using a digital caliper by a blinded observer. The largest (length) and smallest (width) diameters of each lesion were recorded, and the lesion area was calculated using the ellipse area formula. Quantitative data from the groups treated with polar fractions were then statistically compared with the negative and positive control groups.

### Evaluation of systemic toxicity and hepatic parameters

At the end of the treatment period, mice were euthanized by gradual exposure to 100% carbon dioxide (CO₂) from a compressed gas cylinder [31]. Mice were placed in an empty, air-filled euthanasia chamber, and CO₂ was introduced at a displacement rate of 30–50% of the chamber volume per minute [32]. Immediately after the cessation of respiration (while the heartbeat was still present), cardiac blood samples were collected, which served as the method of euthanasia [33]. The blood was then centrifuged to obtain serum, and the levels of seven liver biochemical parameters—including total protein (TP), albumin (Alb), alkaline phosphatase (ALP), aspartate aminotransferase (AST), alanine aminotransferase (ALT), total bilirubin (T. Bili), and direct bilirubin (D. Bili)—were measured.

### Assessment of parasite load in skin lesions

At the end of the treatment period, biopsy specimens were collected from the margins of skin lesions. The specimens were smeared on glass slides, fixed with methanol, and stained with Giemsa. The amastigote load was then assessed under a light microscope. Parasite loads were semi-quantitatively graded as follows: +1 (1–10 amastigotes/100 high-power fields [HPF]), +2 (1–10 amastigotes/10 HPF), +3 (1–10 amastigotes/1 HPF), +4 (10–100 amastigotes/1 HPF), +5 (100–1000 amastigotes/1 HPF), and +6 (> 1000 amastigotes/1 HPF).

### Data analysis

Since the data were not normally distributed, non-parametric tests were used for statistical comparisons. Results are expressed as mean ± standard deviation (SD). For in vitro data, GraphPad Prism version 8 was used to calculate CC₅₀ values. In vivo data were analyzed using SPSS version 26. The Kruskal–Wallis test was used for overall comparisons among multiple groups, and the Mann–Whitney *U* test was applied for pairwise comparisons between treatment groups and control groups. *P* values ≤ 0.05 were considered statistically significant. These statistical methods were used to analyze lesion area (Table 1), parasite load (Table 2), and liver biochemical parameters (TP, Alb, ALP, AST, ALT, T. Bili, and D. Bili; Table 3).

**Table 1:** Mean lesion area (mm^2^) before and after treatment, and statistical comparison between treatment groups and control groups

Group	Mean lesion size (mm²)		*P*-value	
	Before treatment	After treatment	Negative control	Positive control
Negative control	7.22 ± 1.23	27.12 ± 0.26	–	–
Positive control	7.83 ± 0.07	13.53 ± 2.65	–	–
80 mg/kg *A. blitoides*	7.94 ± 1.2	16.57 ± 2.19	0.05	0.05
40 mg/kg *A. blitoides*	7.65 ± 0.22	12.68 ± 2.61	0.05	0.82
20 mg/kg *A. blitoides*	7.45 ± 1.29	13.44 ± 2.61	0.05	0.51
80 mg/kg *C. platycarpos*	7.11 ± 1.73	19.66 ± 2.65	0.05	0.05
40 mg/kg *C. platycarpos*	7.41 ± 1.13	16.05 ± 2.66	0.05	0.51
20 mg/kg *C. platycarpos*	7.96 ± 1.65	5.31 ± 1.88	0.046	0.046
80 mg/kg *C. flava*	7.80 ± 1.78	17.86 ± 3.02	0.05	0.05
40 mg/kg *C. flava*	7.78 ± 1.4	23.69 ± 2.94	0.05	0.05
20 mg/kg *C. flava*	7.01 ± 1.68	6.6 ± 1.6	0.05	0.05
80 mg/kg Mix	7.89 ± 1.5	6.8 ± 2	0.05	0.05
40 mg/kg Mix	7.6 ± 0.32	13.31 ± 1.87	0.05	0.82
20 mg/kg Mix	7.53 ± 0.78	11.55 ± 2.79	0.05	0.27

Data are presented as mean ± SD. The “Before treatment” column represents the mean lesion area (mm^2^) at day 0, prior to treatment initiation

The “After treatment” column represents the mean lesion area (mm^2^) at day 28, one week after the end of treatment

The *p*-values in the “Negative control” and “Positive control” columns were obtained from the Mann–Whitney *U* test comparing each treatment group with the negative and positive control groups, respectively

A *p*-value ≤ 0.05 was considered statistically significant

**Table 2:** Parasite load in treatment and control groups, and p-values from comparing treatment groups with negative and positive control groups

Group	*P*-value		
	Parasite load	Negative control	Positive control
Negative control	+6	–	–
Positive control	+4	–	–
80 mg/kg *A. blitoides*	+5	0.003	0.003
40 mg/kg *A. blitoides*	+3	0.003	0.003
20 mg/kg *A. blitoides*	+4	0.003	1
80 mg/kg *C. platycarpos*	+4	0.003	1
40 mg/kg *C. platycarpos*	+6	1	0.003
20 mg/kg *C. platycarpos*	+5	0.003	0.003
80 mg/kg *C. flava*	+5	0.003	0.003
40 mg/kg *C. flava*	+6	1	0.003
20 mg/kg *C. flava*	+6	1	0.003
80 mg/kg Mix	+4	0.003	1
40 mg/kg Mix	+4	0.003	1
20 mg/kg Mix	+5	0.003	0.003

Data are presented as parasite load scores ranging from +3 to +6

The “Negative control” and “Positive control” columns represent *p*-values obtained from the Mann–Whitney *U* test comparing each treatment group with the negative and positive control groups, respectively

A *p*-value ≤ 0.05 was considered statistically significant

**Table 3:** Average liver biochemical parameters in positive control, negative control, and treatment groups after the treatment period

Group	TP (g/dL)	Alb (g/dL)	ALP (IU/L)	AST (IU/L)	ALT (IU/L)	T. Bili (mg/dL)	D. Bili (mg/dL)
Negative control	5.31 ± 0.04	2.48 ± 0.02	128 ± 11.31	96 ± 13.98	40 ± 7.8	0.16 ± 0.02	0.07 ± 0.01
Positive control	5.73 ± 0.05	2.93 ± 0.07	130 ± 14.15	99 ± 15.23	42 ± 8	0.17 ± 0.02	0.08 ± 0.01
80 mg/kg *A. blitoides*	5.74 ± 0.04	2.68 ± 0.02	131 ± 15.73	99 ± 10.23	42 ± 12.23	0.19 ± 0.03	0.09 ± 0.02
40 mg/kg *A. blitoides*	5.68 ± 0.04	2.68 ± 0.06	131 ± 13.89	98 ± 8.44	42 ± 10.1	0.18 ± 0.05	0.08 ± 0.05
20 mg/kg *A. blitoides*	5.6 ± 0.06	2.66 ± 0.03	130 ± 11.25	98 ± 10.96	41 ± 11.38	0.18 ± 0.03	0.08 ± 0.01
80 mg/kg *C. platycarpos*	5.65 ± 0.05	2.55 ± 0.02	130 ± 11.34	98 ± 13.6	41 ± 12.33	0.19 ± 0.06	0.09 ± 0.05
40 mg/kg *C. platycarpos*	5.64 ± 0.04	2.52 ± 0.05	129 ± 10.76	98 ± 8.98	40 ± 10.71	0.19 ± 0.04	0.09 ± 0.01
20 mg/kg *C. platycarpos*	5.62 ± 0.07	2.49 ± 0.03	129 ± 10.14	97 ± 11.54	40 ± 6.1	0.19 ± 0.03	0.08 ± 0.02
80 mg/kg *C. flava*	5.72 ± 0.06	2.67 ± 0.02	130 ± 12.67	96 ± 12.32	41 ± 10.51	0.19 ± 0.06	0.09 ± 0.05
40 mg/kg *C. flava*	5.68 ± 0.05	2.66 ± 0.06	128 ± 9.56	96 ± 11.6	41 ± 14.54	0.18 ± 0.02	0.09 ± 0.02
20 mg/kg *C. flava*	5.65 ± 0.02	2.56 ± 0.04	128 ± 13.52	96 ± 9.89	41 ± 8.05	0.16 ± 0.01	0.09 ± 0.01
80 mg/kg Mix	5.33 ± 0.04	2.71 ± 0.02	131 ± 14.16	97 ± 7.07	41 ± 7.74	0.19 ± 0.05	0.09 ± 0.03
40mg/kg Mix	5.32 ± 0.06	2.69 ± 0.05	131 ± 12.3	97 ± 10.4	40 ± 8.5	0.17 ± 0.02	0.07 ± 0.08
20 mg/kg Mix	5.32 ± 0.03	2.7 ± 0.03	129 ± 10.67	96 ± 8.32	40 ± 7.35	0.17 ± 0.05	0.07 ± 0.02

Data are presented as mean ± SD

*TP* total protein, *Alb* albumin, *ALP* alkaline phosphatase, *AST* aspartate aminotransferase, *ALT* alanine aminotransferase, *T. Bili* total bilirubin, *D. Bili* direct bilirubin

Units: *g/dL* gram per deciliter, *IU/L* international unit per liter, *mg/dL* milligram per deciliter

Statistical analysis was performed using the Mann–Whitney *U* test comparing each treatment group with the negative control group. No statistically significant differences were observed between the negative control group and any of the treatment groups or the positive control group for any of the parameters (*p* > 0.05)

## Results

### Antileishmanial activity of the polar fractions

**Fig. 1:**
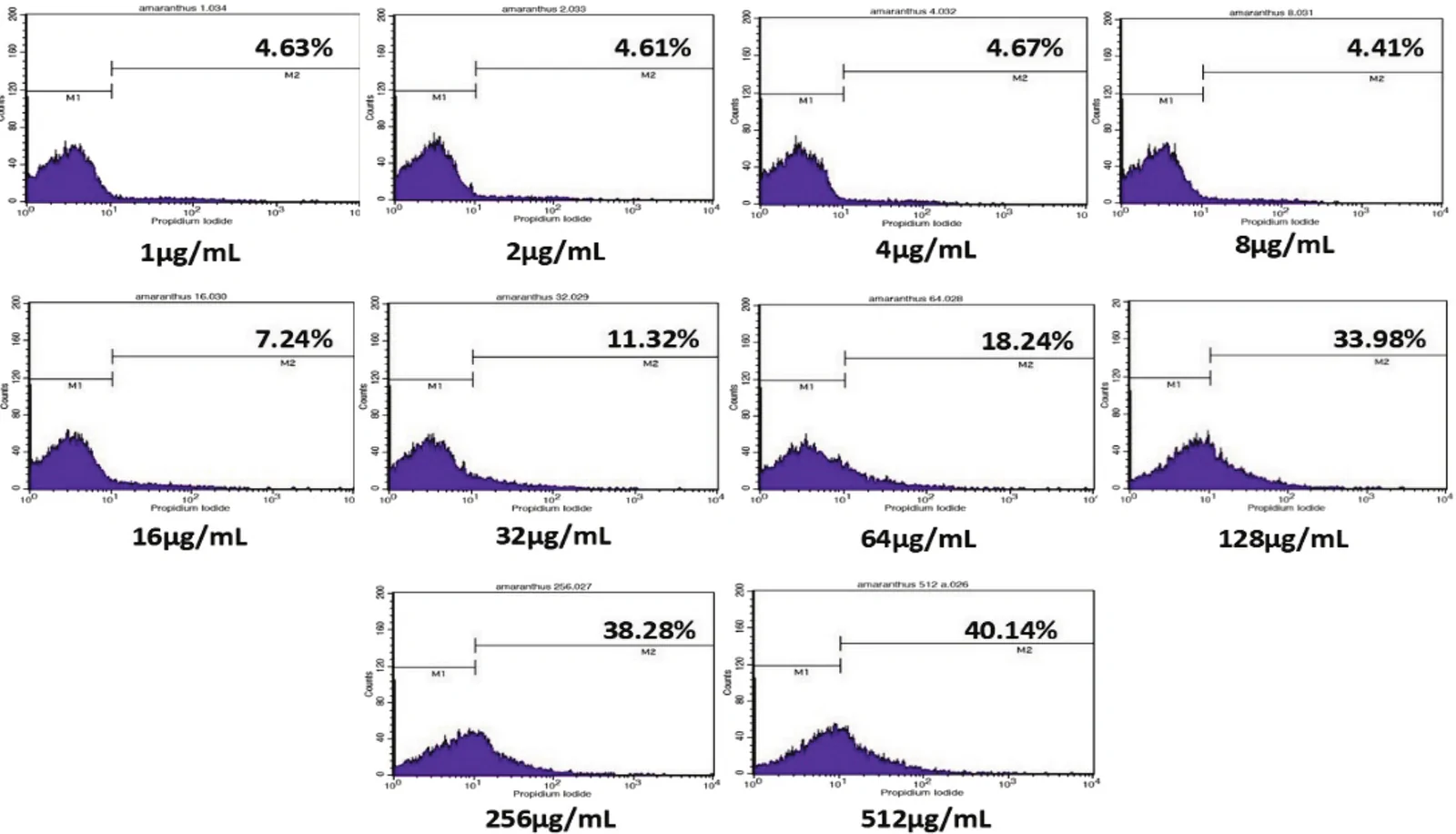
Flow cytometry results of *L. major* promastigotes exposed to different concentrations of *A. blitoides *polar fraction (1 to 512 μg/mL)

**Fig. 2:**
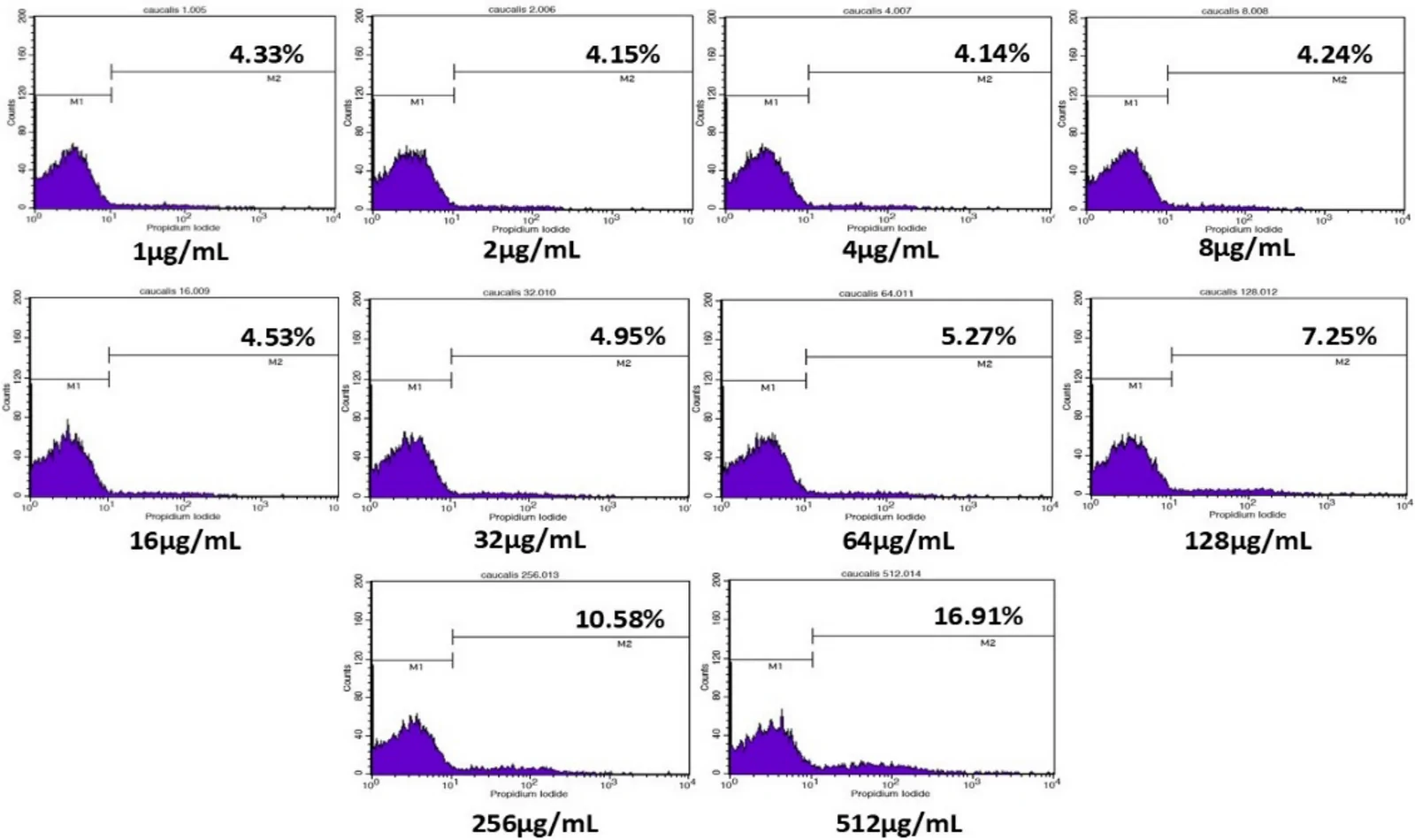
Flow cytometry results of *L. major* promastigotes exposed to different concentrations of *C. platycarpos* polar fraction (1 to 512 μg/mL)

**Fig. 3:**
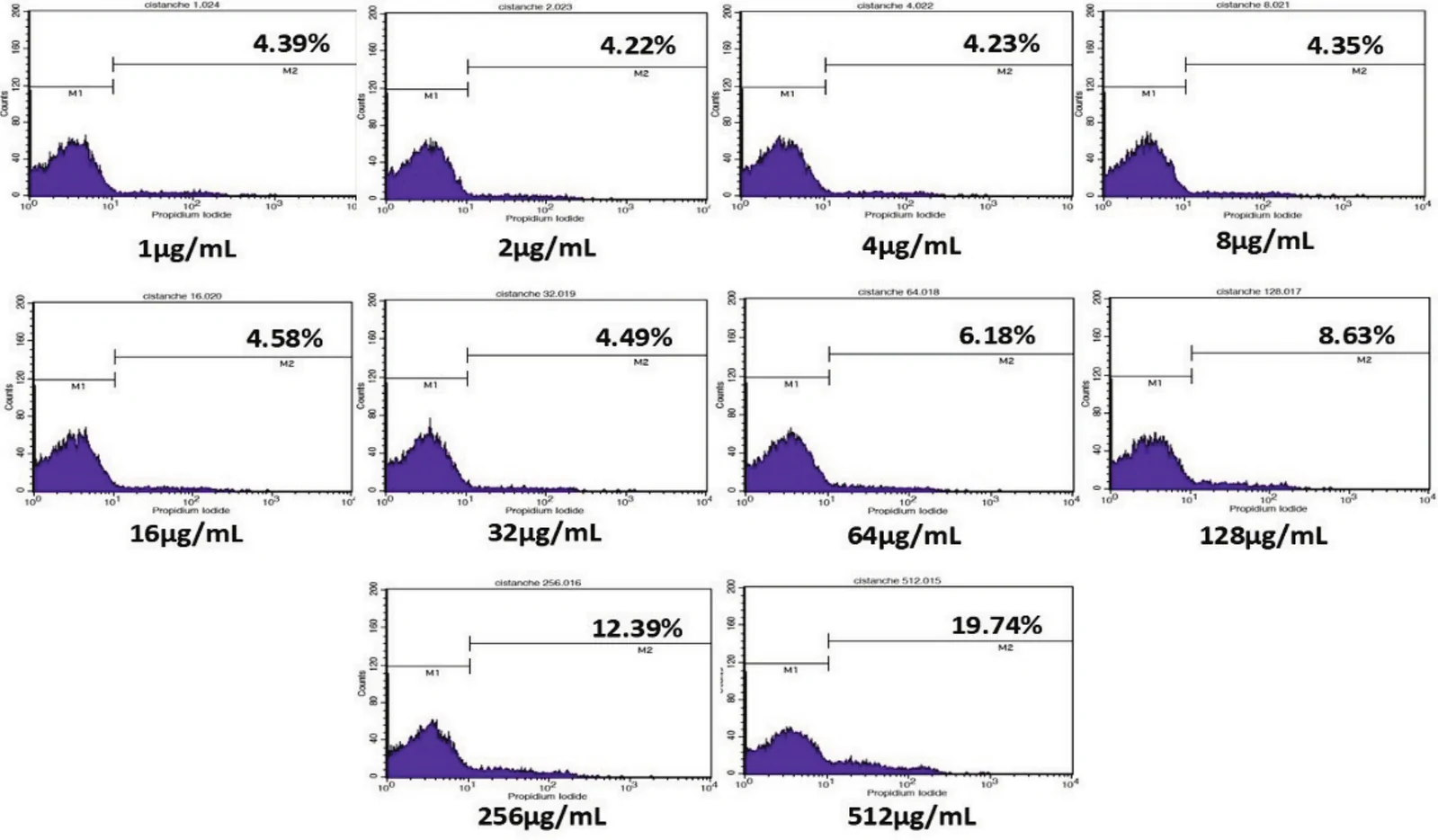
Flow cytometry results of *L. major* promastigotes exposed to different concentrations of *C. flava* polar fraction (1 to 512 μg/mL)

**Fig. 4:**
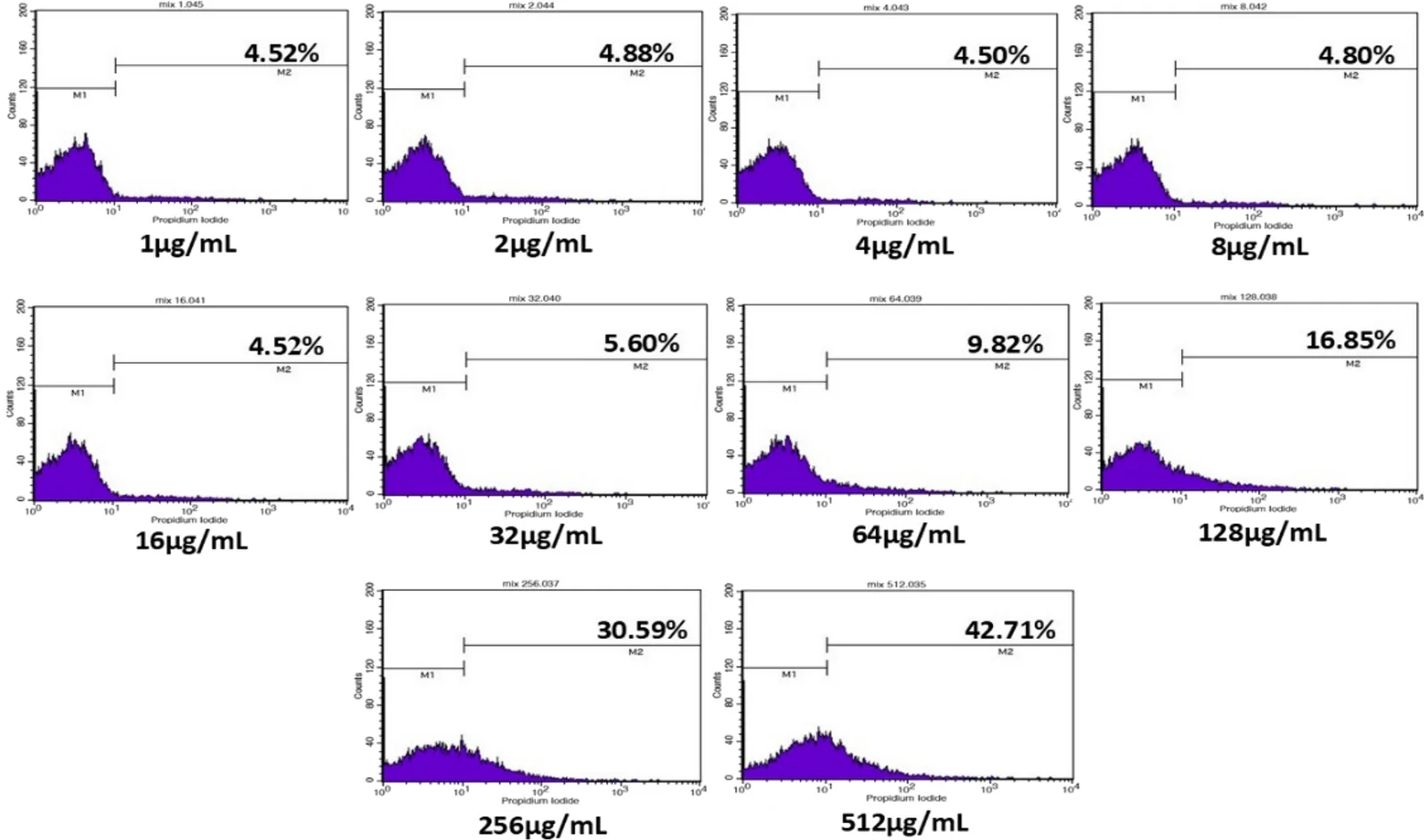
Flow cytometry results of *L. major* promastigotes exposed to different concentrations of a mixture of three polar fractions (1 to 512 μg/mL).

**Fig. 5:**
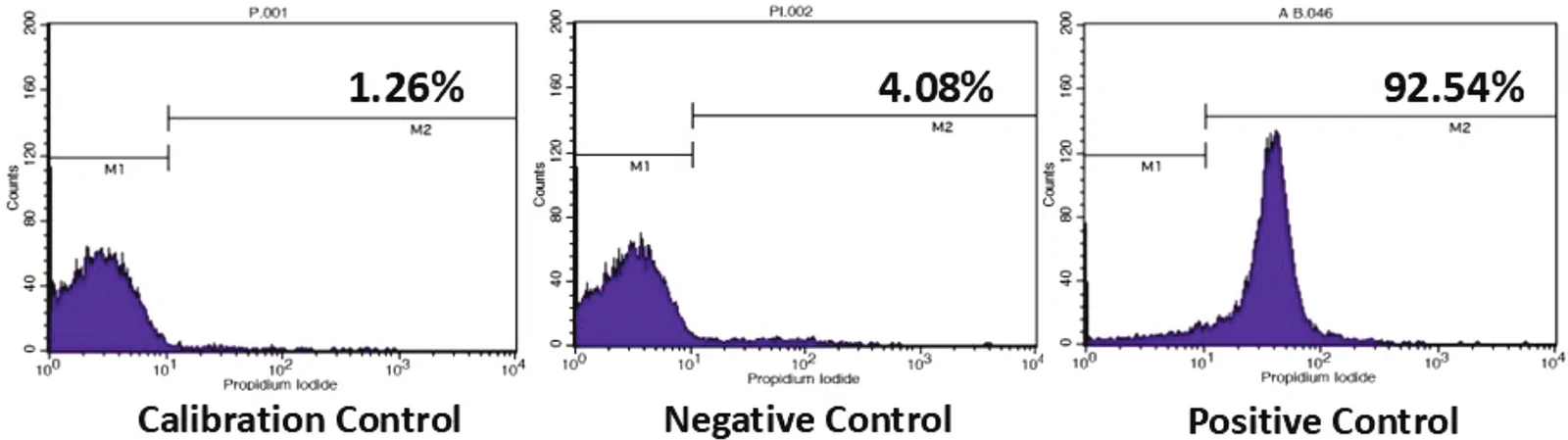
Flow cytometry results of positive, negative and calibration controls of *L. major* promastigotes

**Fig. 6:**
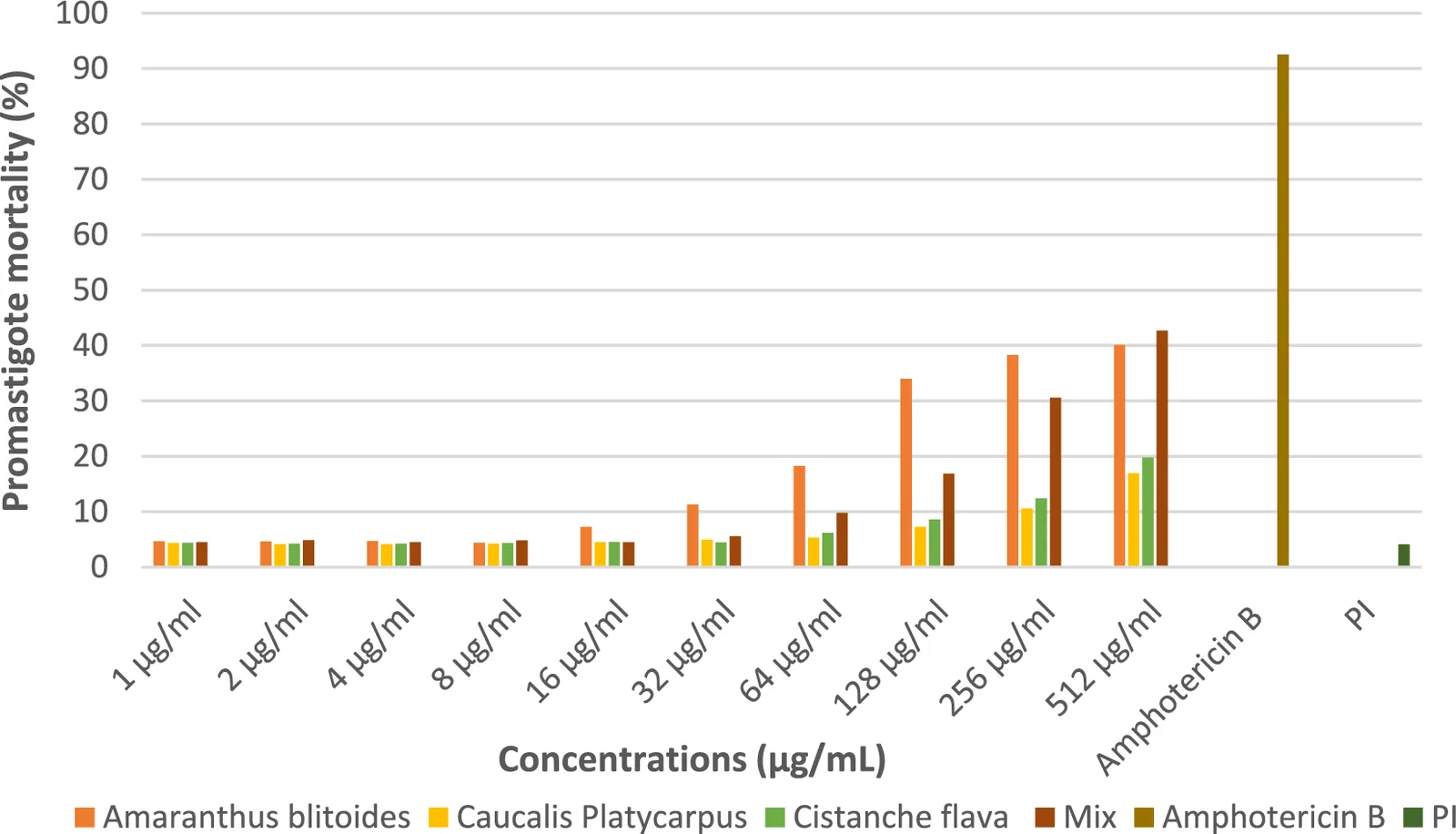
Comparison of promastigote mortality by flow cytometry exposed to different concentrations of polar fractions (1 to 512 μg/mL)

Flow cytometric analysis revealed an increasing trend in mortality for all polar fractions with rising concentrations. The mortality rates of *A. blitoides* at concentrations of 1, 2, 4, 8, 16, 32, 64, 128, 256, and 512 μg/mL were 4.63, 4.61, 4.67, 4.41, 7.24, 11.32, 18.24, 33.98, 38.28, and 40.14%, respectively (Fig. 1). The mortality rates of *C. platycarpos* at the same concentrations were 4.33, 4.15, 4.14, 4.24, 4.53, 4.95, 5.27, 7.25, 10.58, and 16.91%, respectively (Fig. 2). The mortality rates of *C. flava* at the same concentrations were 4.39, 4.22, 4.23, 4.35, 4.58, 4.49, 6.18, 8.63, 12.39, and 19.74%, respectively (Fig. 3). The mortality rates of the mixture of the three polar fractions at the same concentrations were 4.52, 4.88, 4.50, 4.80, 4.52, 5.60, 9.82, 16.85, 30.59, and 42.71%, respectively (Fig. 4). The mortality rates for the negative, positive, and calibration controls were 4.08, 92.54, and 1.26%, respectively (Fig. 5). Figure 6 compares the percentage mortality of promastigotes exposed to different concentrations of the polar fractions, as assessed by flow cytometry.

### Cytotoxic effects of the polar fractions on macrophages

**Fig. 7:**
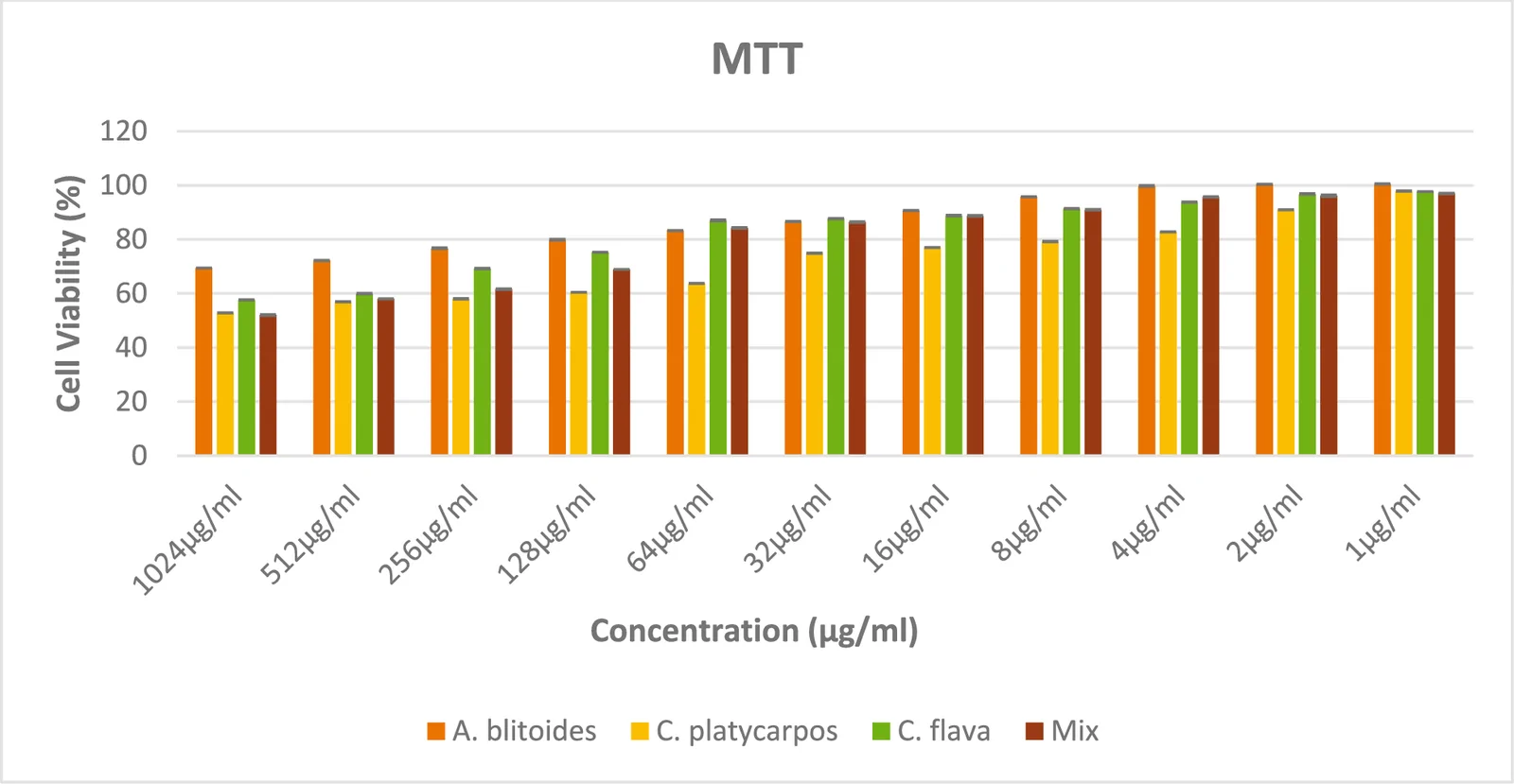
Comparison of the percentage of macrophage viability by MTT method exposed to different concentrations of polar fractions (1 to 1024 μg/ml)

Macrophage viability was assessed after 48 h of exposure to different concentrations (1 to 1024 μg/mL) of the polar fractions and their mixture (Fig. 7). A progressive reduction in cell viability was observed with increasing concentrations of all tested polar fractions, with the most pronounced cytotoxic effect detected at the highest concentration (1024 μg/mL). The CC₅₀ values for *A. blitoides*, *C. platycarpos*, *C. flava*, and their mixture were determined to be 5150, 850.8, 1549, and 945.7 μg/mL, respectively. Among the tested polar fractions, *A. blitoides* exhibited the lowest cytotoxicity (highest CC₅₀), while *C. platycarpos* showed the highest cytotoxicity (lowest CC₅₀).

### Evaluation of lesion size changes following treatment

**Fig. 8:**
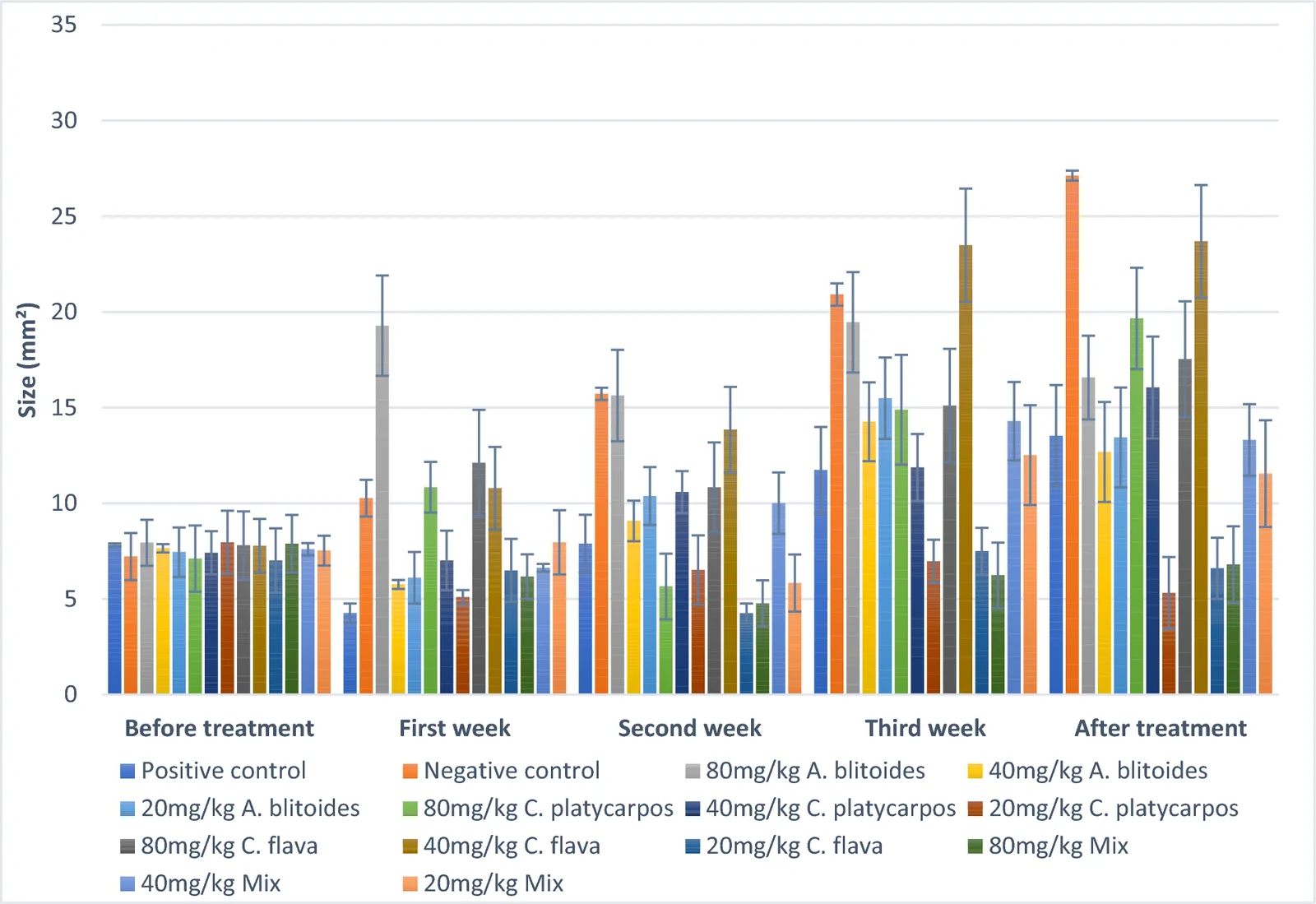
Changes in mean lesion area (mm^2^) in different treatment groups during the treatment period

According to the results of the Kruskal–Wallis test, no significant difference was observed in the mean lesion area among the groups before treatment initiation (*p* > 0.05). Figure 8 illustrates the trend of changes in mean lesion area during the treatment period, and Table 1 presents the mean ± SD of lesion area (mm^2^) before and after treatment, along with the p-values obtained from the Mann–Whitney *U* test comparing each treatment group with the control groups. In the negative control group, the mean lesion area increased from 7.22 ± 1.23 mm^2^ to 27.12 ± 0.26 mm^2^. Similarly, in the positive control group, the mean lesion area increased from 7.83 ± 0.07 mm^2^ to 13.53 ± 2.65 mm^2^. A reduction in mean lesion area at the end of the treatment period compared to baseline was observed in three groups: 20 mg/kg *C. platycarpos* (from 7.96 ± 1.65 to 5.31 ± 1.88 mm^2^), 20 mg/kg *C. flava* (from 7.01 ± 1.68 to 6.6 ± 1.6 mm^2^), and 80 mg/kg Mix (from 7.89 ± 1.5 to 6.8 ± 2 mm^2^). This reduction was statistically significant compared to both the negative and positive control groups (*p* ≤ 0.05; Table 1). Clinically, only in these three groups did inflammation and redness diminish compared to baseline, and superficial crusting was observed on the lesions. In the remaining treatment groups, the mean lesion area increased by the end of the treatment period. However, despite this increase, the final lesion size in all these groups was significantly smaller than that of the negative control group (*p* = 0.05; Table 1). The final lesion areas in these groups ranged from 11.55 ± 2.79 mm^2^ to 23.69 ± 2.94 mm^2^ (Table 1). Regarding the positive control group, the final lesion areas in the groups treated with 40 mg/kg *A. blitoides*, 20 mg/kg *A. blitoides*, 40 mg/kg Mix, and 20 mg/kg Mix were 12.68 ± 2.61, 13.44 ± 2.61, 13.31 ± 1.87, and 11.55 ± 2.79 mm^2^, respectively. Although these values were smaller than that of the positive control group, the differences were not statistically significant (*p* > 0.05; Table 1). In contrast, the groups treated with 80 mg/kg *A. blitoides*, 80 mg/kg *C. platycarpos*, 80 mg/kg *C. flava*, and 40 mg/kg *C. flava* showed significantly larger lesion areas, with values of 16.57 ± 2.19, 19.66 ± 2.65, 17.86 ± 3.02, and 23.69 ± 2.94 mm^2^, respectively, compared to the positive control group (p = 0.05; Table 1). Moreover, the 40 mg/kg *C. platycarpos* group also exhibited a larger lesion area of 16.05 ± 2.66 mm^2^ compared to the positive control group; however, this difference was not statistically significant (*p* > 0.05; Table 1).

### Assessment of parasite load after treatment

Parasite load in the treatment groups was compared with the negative and positive control groups using the Mann–Whitney *U* test, as presented in Table 2. The negative control group showed a parasite load of +6, while the positive control group showed a parasite load of +4. The parasite load of the treatment groups ranged from +3 to +6. The 40 mg/kg *A. blitoides* group showed the lowest parasite load (+ 3) among all treatment groups, which was significantly lower than both the negative and positive control groups (*p* = 0.003). The 20 mg/kg *A. blitoides*, 80 mg/kg  *C. platycarpos*, 80 mg/kg Mix, and 40 mg/kg Mix groups showed a parasite load of +4. These groups had significantly lower parasite loads compared to the negative control (*p* = 0.003), and were not significantly different from the positive control (*p* = 1). The 80 mg/kg *A. blitoides*, 20 mg/kg  *C. platycarpos*, 80 mg/kg  *C. flava*, and 20 mg/kg Mix groups showed a parasite load of +5. All these groups had significantly lower parasite loads compared to the negative control (*p* = 0.003), but significantly higher parasite loads compared to the positive control (*p* = 0.003). The 40 mg/kg  *C. platycarpos*, 40 mg/kg  *C. flava*, and 20 mg/kg  *C. flava* groups showed a parasite load of +6. These groups were not significantly different from the negative control (*p* = 1), but had significantly higher parasite loads compared to the positive control (*p* = 0.003).

### Average liver biochemical parameters after treatment

Serum levels of liver biochemical parameters were measured at the end of the treatment period to assess systemic toxicity. According to the results of the Mann–Whitney *U* test, there was no statistically significant difference between the negative control group and each of the treatment groups, as well as the positive control group (*p* > 0.05). Table 3 presents the mean ± SD values of TP, Alb, ALP, AST, ALT, T. Bili, and D. Bili in the positive control group, the negative control group, and the groups treated with polar fractions.

## Discussion

### Background and therapeutic potential of medicinal plants against CL

CL is recognized as a research priority by the WHO [34]. Treatment of this disease remains challenging, and medicinal plants are considered valuable resources for the development of new therapies due to their bioactive compounds [35]. Previous studies have suggested the positive effects of plant extracts in the treatment of CL. For example, Sharifi-Rad et al. investigated the extract of *Veronica persica* Poir for the treatment of CL caused by *L. major* and reported that this extract exhibited antileishmanial activity against *L. major* promastigotes and accelerated the healing of skin lesions in BALB/c mice [36]. In another study, Kheirandish et al. stated that olive leaf extract showed significant therapeutic effects against CL caused by *L. major*, reducing the size of skin lesions in BALB/c mice [37].

### Therapeutic potential of polar plant extracts

The therapeutic potential of polar plant extracts has been highlighted in several studies due to the presence of effective bioactive compounds. For instance, Ri et al. reported that extracts obtained using polar solvents from *Aurea Helianthus* flowers were rich in flavonoids and phenolic compounds and demonstrated significant antioxidant activity [38]. In another study by Yusnawan, the polar extract of *Ageratum conyzoides* leaves was found to contain alkaloids, flavonoids, tannins, saponins, and terpenoids, with polar solvent extraction yielding more diverse secondary metabolites compared to non-polar solvents [39]. Vyshnavi et al. pointed out the therapeutic potential of medicinal plants for various diseases, noting that tannins and saponins possess antioxidant and anti-inflammatory effects [40]. G/giorgis et al. reported that the 80% methanolic crude extract and solvent fractions of *Justicia schimperiana* leaves promoted wound healing in mice [41]. Lee et al. noted that methanolic and aqueous polar extracts contain phenolic and flavonoid bioactive compounds, and flavonoids may regulate inflammatory pathways through antioxidant activity [16]. Furthermore, Fioroni et al. reported that polar extracts exhibited 2- to 18-fold higher antioxidant activity compared to non-polar extracts, attributing this superiority to the higher levels of polyphenolic and flavonoid compounds present in polar extracts [42].

### Plant selection and bioactive compounds

Based on previous studies and considering the importance of bioactive compounds, three plants—*A. blitoides*, *C. platycarpos*, and *C. flava*—were selected for investigation in this study. This selection was based on evidence suggesting that bioactive compounds present in plant extracts may exert significant anti-inflammatory, antioxidant, and antimicrobial effects. Many of the polar phenolic compounds, such as flavonoid or phenolic glycosides, exhibited significant antileishmanial and anti-inflammatory activities in in vitro or animal models [43–45]. Using our fractionation method, we aimed to concentrate such metabolites into a polar phenolic fraction [46]. Accordingly, we focused our research on fractions that may contain a high level of these compounds to investigate their possible antileishmanial activity [23, 47–51]. *Amaranthus* species have been shown to possess antioxidant, anti-inflammatory, and antimicrobial activities in numerous studies and contain a variety of nutrients and bioactive compounds, including pigments, vitamin C, phenols, flavonoids, saponins, hemagglutinins, phytins, nitrates, and oxalates. Among these, saponins, protoalkaloids, and betacyanins are believed to be responsible for the medicinal effects of the plant, while polyphenols and flavonoids may accelerate wound healing [17, 52]. Similarly, *Cistanche* species contain phenylethanoid glycosides (PhGs) and iridoids, which have been reported to possess anti-inflammatory, immunomodulatory, and hepatoprotective effects in several studies [19–22]. Thus, the presence of these compounds in *Cistanche* likely plays an important role in modulating the host immune response and reducing inflammation. Additionally, *Caucalis platycarpos* contains significant phenolic and flavonoid content, with caffeic acid and luteolin reported as its dominant components. These compounds are known to have potent anti-inflammatory and antioxidant effects [23, 24, 49] and may therefore be effective in reducing inflammation and supporting tissue repair.

### Study objectives

Considering that previous studies have indicated that effective bioactive compounds in plant extracts are mainly concentrated in polar fractions, we aimed to evaluate the effects of polar fractions derived from methanolic extracts of the three mentioned plants—individually and as a mixture—on the improvement of skin lesions caused by *L. major*, which had not been investigated previously.

### In vitro evaluation

Given that one of the main criteria for a drug to advance to the in vivo phase is its evaluation in vitro, the in vitro activities of the polar fractions were initially assessed. Flow cytometry was employed to investigate the antileishmanial effect, and the MTT assay was used to evaluate the cytotoxicity of the polar fractions. The flow cytometric analysis revealed that increasing concentrations led to increased mortality in all tested polar fractions. Among the individual fractions, *A. blitoides* exhibited the highest antileishmanial activity, with mortality rates ranging from 4.63% at 1 μg/mL to 40.14% at 512 μg/mL (Fig. 1). In contrast, *C. platycarpos* and *C. flava* showed lower mortality rates, reaching 16.91% and 19.74% at the highest concentration, respectively (Figs. 2 and 3). Notably, the mixture of the three polar fractions demonstrated the highest overall activity, with 42.71% mortality at 512 μg/mL, suggesting a potential synergistic effect among the bioactive compounds present in the different plants (Fig. 4). The enhanced activity of the mixture may be attributed to the complementary mechanisms of action of the phytochemicals, which could collectively target multiple pathways involved in parasite survival. The positive control (amphotericin B) exhibited 92.54% mortality, confirming the reliability of the assay. The negative and calibration controls showed minimal mortality (4.08% and 1.26%, respectively), indicating that the observed effects were specifically due to the treatments (Fig. 5). Regarding cytotoxicity, the MTT assay results demonstrated that the polar fractions exhibited low toxicity against murine macrophages (Fig. 7). The CC₅₀ values for *A. blitoides*, *C. platycarpos*, *C. flava*, and the mixture were 5150, 850.8, 1549, and 945.7 μg/mL, respectively. These values are considerably higher than the concentrations required for antileishmanial activity (512 μg/mL), indicating a favorable selectivity index. Among the tested fractions, *A. blitoides* showed the lowest cytotoxicity (highest CC₅₀), while *C. platycarpos* exhibited the highest cytotoxicity (lowest CC₅₀). The relatively high CC₅₀ values suggest that the polar fractions possess a favorable safety profile, which is a critical consideration for the development of new therapeutic agents. The lack of significant cytotoxicity against macrophages, combined with their antileishmanial activity, supports the potential of these polar fractions for further in vivo evaluation.

### In vivo efficacy, lesion size reduction, and parasite load

Following the analysis of in vitro results, the polar fractions proceeded to the in vivo phase. In the in vivo study, the negative control group showed a gradual increase in lesion size, indicative of the natural progression of CL, which was consistent with the high parasite load observed in this group (+ 6). These findings highlight the lack of effective treatment in this group and the natural disease course. In the positive control group, despite treatment with the standard drug, an initial decrease followed by a limited increase in lesion size was observed, with a final parasite load of +4. Although this was lower than that of the negative control, it remained considerable. This limited response may be attributed to factors such as restricted drug penetration into the lesion area, local tissue alterations, or reduced blood supply; however, further investigation is required to confirm these possibilities. Among the treatment groups, only those receiving 20 mg/kg *C. platycarpos*, 20 mg/kg *C. flava*, and 80 mg/kg Mix exhibited a reduction in lesion size compared to baseline (Fig. 8; Table 1). At the end of the treatment period, the lesion size in these three groups was significantly smaller than that of both the negative and positive control groups (*p* ≤ 0.05; Table 1). Clinical observations also revealed decreased local inflammation and redness in these three groups, suggesting that this improvement may be attributed to the formation of a natural crust over the lesion and the initiation of tissue repair processes. Interestingly, despite the reduction in lesion size and inflammation, the parasite load in these three groups was +5 for 20 mg/kg *C. platycarpos*, +6 for 20 mg/kg *C. flava*, and +4 for 80 mg/kg Mix (Table 2). These observations suggest that the reduction in local inflammation and lesion size may not necessarily be accompanied by a proportional decrease in parasite load, and might reflect early stages of tissue repair rather than complete parasite elimination. One possible explanation for this dissociation is that the polar fractions may exert their effects primarily through modulation of the host immune response, rather than through direct antiparasitic activity. Accordingly, reducing local inflammation—even in the presence of viable parasites—may lead to significant lesion shrinkage and clinical improvement. Furthermore, by downregulating local inflammatory responses, these fractions may limit tissue damage and facilitate healing, even without considerably affecting parasite viability. On the other hand, the persistence of parasites despite clinical improvement highlights the complexity of host–parasite interactions and emphasizes the importance of evaluating treatment efficacy based on both clinical and parasitological criteria. Nevertheless, this interpretation remains speculative and requires confirmation through direct measurement of cytokines and immune cell populations in future studies. In the remaining treatment groups, the mean lesion area increased during the treatment period; however, lesions remained smaller than those in the negative control group. This finding suggests that treatment with the studied polar fractions may have partially inhibited lesion progression and exerted a relative protective effect. In four treatment groups—40 mg/kg *A. blitoides*, 20 mg/kg *A. blitoides*, 40 mg/kg Mix, and 20 mg/kg Mix—the mean lesion area was slightly smaller than that of the positive control group; however, these differences were not statistically significant. The parasite load in these groups was +3, +4, +4, and +5, respectively, with the 40 mg/kg *A. blitoides* group showing the lowest parasite load among all treatment groups (Table 2). These findings highlight a critical dissociation between clinical and parasitological outcomes, demonstrating that lesion size may increase even when parasite load is reduced. This observation underscores the limitation of relying solely on clinical appearance as a surrogate marker of therapeutic efficacy and reinforces the necessity of incorporating direct parasitological assessment in the evaluation of treatment response.

### Liver biochemical parameters and safety

The evaluation of liver biochemical parameters is critical for assessing the potential toxicity of the polar fractions. In this study, the lack of significant changes in serum levels of TP, Alb, ALP, AST, ALT, T. Bili, and D. Bili compared to the control groups (*p* > 0.05; Table 3) indicates that the polar fractions did not adversely affect hepatic function or structural integrity. The stability of ALT and AST levels is particularly noteworthy, as these enzymes are sensitive markers of hepatocellular damage [53]. Their normal range across all treatment groups suggests that the polar fractions did not cause hepatocyte necrosis or dysfunction. Similarly, the maintenance of normal ALP levels reflects the absence of biliary obstruction or cholestasis [54]. Unchanged levels of total protein and albumin further support the preservation of hepatic synthetic function, as these parameters are key indicators of the liver’s ability to produce essential plasma proteins [55]. Additionally, normal bilirubin levels rule out clinically significant cholestasis or hemolysis [56]. Collectively, these findings indicate a favorable safety profile for the polar fractions at the tested doses, which is a critical consideration for their potential therapeutic application. However, while these results are promising, long-term toxicity studies in other animal models are required to confirm the safety of these fractions before clinical application.

### Comparison with previous studies

The present findings are consistent with previous studies demonstrating the therapeutic potential of polar plant extracts against CL. Several studies have reported that plant extracts rich in bioactive compounds can reduce lesion size and modulate the host immune response. For example, Maleki et al. reported that the hydroalcoholic extract of leaves, roots, and seeds of *Arctium lappa* significantly reduced lesion size in mice infected with *L. major*. They attributed this reduction to the combined effects of the extract on multiple mechanisms, including anti-inflammatory activities and induction of host immune responses, while noting that apparent improvement does not necessarily indicate complete parasite eradication [57]. This observation is in line with our findings. In our study, lesion size reduction was not accompanied by a corresponding decrease in parasite load. This supports the hypothesis that the therapeutic effects of the polar fractions may be primarily mediated through immune modulation, rather than direct antiparasitic activity. Similarly, Badrizadeh et al. stated that the aqueous extract of *Urtica dioica* has antileishmanial effects and reported that the lesion size of BALB/c mice infected with *L. major* in the groups treated with this plant extract was significantly smaller than in the untreated groups. They suggested that the extract likely reduced local inflammation by modulating the host immune system and altering cytokine profiles (increasing IFN-γ and decreasing IL-4), thereby contributing to reduced apparent lesion size without completely eliminating the parasite [58]. This is consistent with our hypothesis that the polar fractions may modulate the host immune response, contributing to reduced lesion size and inflammation without achieving uniform parasite clearance. Furthermore, Gholamrezaei et al. showed that aqueous and alcoholic extracts of *Foeniculum vulgare* seeds possessed antileishmanial effects, reducing parasite load in lesions and spleens after four weeks of treatment. However, changes in lesion size were not significant in some groups and, in certain cases, did not differ from the positive control. They attributed this finding to the point that although the active compounds in the extract can reduce the parasite load, their effect on lesion shrinkage may be limited or that more time may be required for complete recovery [59]. This finding is consistent with our observation that apparent lesion size does not always reflect the actual parasite load, emphasizing the importance of evaluating both clinical and parasitological parameters. Additionally, Amrati et al. reported that the polyphenolic extract of *Caralluma europaea* contains flavonoids and phenolic acids, noting that compounds such as kaempferol, luteolin, and ferulic acid play important roles in wound healing. They stated that kaempferol possesses antioxidant and anti-inflammatory properties that may accelerate wound healing and closure; luteolin may modulate inflammatory and antioxidant activities; and ferulic acid appears effective in reducing wound area [60]. Based on previous reports, it is possible that similar bioactive compounds may be present in our polar fractions and could contribute to the observed reduction in lesion size and inflammation, supporting the potential role of phenolic and flavonoid compounds in wound healing and tissue repair. However, direct phytochemical characterization is needed to confirm the presence of these compounds. Collectively, these comparisons suggest that our findings are in line with previous research, possibly highlighting the role of polar plant extracts in immune modulation and lesion improvement, while emphasizing the importance of evaluating both clinical and parasitological parameters in the assessment of treatment efficacy.

### Novelty and significance of the study

This is the first study to evaluate the antileishmanial activity of polar fractions from *A. blitoides*, *C. platycarpos*, and *C. flava*, individually and as a mixture, in both in vitro and in vivo models. Our findings indicate a dissociation between lesion size reduction and parasite clearance, which raises the possibility that immune modulation may play a role in the observed effects. The polar fractions also exhibited a favorable safety profile, as suggested by the absence of hepatotoxicity and low cytotoxicity against macrophages. These preliminary findings highlight the potential of these fractions as complementary candidates for further investigation in the management of CL, though additional studies are needed to confirm their efficacy and safety.

### Limitations and future directions

One of the main limitations of this study is the lack of identification of the specific bioactive compounds present in the polar fractions. Future research focusing on the isolation and chemical characterization of the active constituents, as well as the elucidation of their biological mechanisms, would help to better understand the therapeutic potential of these fractions and support their development as safe and effective candidates for the treatment of CL. In addition, another limitation is the absence of correction for multiple comparisons, which may increase the risk of type I error. Nevertheless, the overall consistency of the observed effects across different parameters supports the reliability of the findings.

## Conclusion

The present study was designed to evaluate the antileishmanial potential of polar fractions from *A. blitoides*, *C. platycarpos*, and *C. flava*, individually and as a mixture, using in vitro and in vivo models. Our findings indicated that these fractions exhibited mild-to-moderate antileishmanial activity against *L. major* promastigotes, as assessed by flow cytometry, with the highest mortality observed at 512 μg/mL. Importantly, the mixture of the three fractions showed superior activity compared to individual fractions, suggesting a possible synergistic effect. Cytotoxicity evaluation using the MTT assay revealed that the polar fractions did not exhibit significant toxicity against murine macrophages, with CC₅₀ values considerably higher than the effective concentrations. In the in vivo model of CL in BALB/c mice, treatment with 20 mg/kg *C. platycarpos*, 20 mg/kg *C. flava*, and 80 mg/kg Mix significantly reduced lesion size and improved local inflammation. However, parasite load remained relatively high in these groups, indicating that lesion improvement was not necessarily associated with parasite clearance. This finding supports the hypothesis that the therapeutic effects may be primarily mediated through immune modulation rather than direct antiparasitic activity. Furthermore, the absence of significant changes in liver biochemical parameters (TP, Alb, ALP, AST, ALT, T. Bili, and D. Bili) supported the favorable safety profile of the tested fractions. These findings suggest that polar fractions from these three plant species possess antileishmanial potential. The scientific contribution of this study lies in the first systematic evaluation of these plants and the possible role of immune modulation. Moreover, the favorable safety profile supports their potential as complementary candidates for CL management. Future research should focus on the identification and characterization of the active compounds responsible for the observed effects, as well as the elucidation of their mechanisms of action. Long-term safety and efficacy studies in other animal models are also warranted to support further development toward clinical application.

## Data Availability

The datasets used and/or analyzed during the current study are available upon reasonable request.

## Abbreviations

Alb: Albumin
ALP: Alkaline phosphatase
ALT: Alanine aminotransferase
AST: Aspartate aminotransferase
CL: Cutaneous leishmaniasis
D. Bili: Direct bilirubin
FBS: Fetal bovine serum
g/dL: Gram per deciliter
HPF: High-power field
IU/L: International unit per liter
mg/dL: Milligram per deciliter
MTT: 3-(4,5-Dimethylthiazol-2-yl)-2,5-diphenyltetrazolium bromide
OD: Optical density
PBS: Phosphate-buffered saline
Pen-Strep: Penicillin–streptomycin
PI: Propidium iodide
SD: Standard deviation
T. Bili: Total bilirubin
TP: Total protein
WHO: World Health Organization
